# Isoflurane Induces Learning Impairment That Is Mediated by Interleukin 1β in Rodents

**DOI:** 10.1371/journal.pone.0051431

**Published:** 2012-12-12

**Authors:** Lin Cao, Liaoliao Li, Daowei Lin, Zhiyi Zuo

**Affiliations:** 1 Department of Anesthesiology, University of Virginia, Charlottesville, Virginia, United States of America; 2 Department of Anesthesiology, Sun-Yat-Sen Memorial Hospital, Sun-Yat-Sen University, Guangzhou, Guangdong, China; Massachusetts General Hospital, United States of America

## Abstract

Postoperative cognitive decline is a clinical syndrome. Volatile anesthetics are commonly used during surgery. It is conceivable that volatile anesthetics may contribute to postoperative cognitive decline. Isoflurane can impair cognitive functions of animals under certain conditions. However, the mechanisms for this impairment are not clear. Here, male 18-month old Fisher 344 rats or 10-week old mice were exposed to 1.2 or 1.4% isoflurane for 2 h. Our studies showed that isoflurane impaired the cognitive functions of the rats in Barnes maze. Isoflurane-exposed rats had reduced freezing behavior during the training sessions in the fear conditioning test. This isoflurane effect was attenuated by lidocaine, a local anesthetic with anti-inflammatory property. Rats that had training sessions and were exposed to isoflurane 30 min later had freezing behavior similar to that of control animals. Isoflurane increased the expression of interleukin 1β (IL-1β), interleukin-6 and activated caspase 3 in the hippocampus of the 18-month old rats. IL-1β positive staining was co-localized with that of NeuN, a neuronal marker. The increase of IL-1β and activated caspase 3 but not interleukin-6 was attenuated by lidocaine. Isoflurane also impaired the cognitive functions of 10-week old C57BL/6J mice and increased IL-1β in their hippocampi. However, isoflurane did not affect the cognitive functions of IL-1β deficient mice. Our results suggest that isoflurane impairs the learning but may not affect the recall of the aged rats. IL-1β may play an important role in this isoflurane effect.

## Introduction

More than 20 million patients undergo surgery under general anesthesia each year in the USA. The majority of these patients receive volatile anesthetics as the primary anesthetics during surgery [1]. This popularity for volatile anesthetics is due to many features of these anesthetics. For example, they are easy to use and have multiple pharmacological effects, such as anesthesia, analgesia and muscle relaxation that are components of general anesthesia. Since they usually are eliminated quickly from the body, it has been assumed that their drug effects disappear soon after their elimination. However, this assumption has been challenged by many lines of evidence. For example, we have shown that exposure to isoflurane, a commonly used volatile anesthetic, for 30 min changes the expression of activated/phosphorylated mitogen-activated protein kinase for many days [2], [3]. Also, volatile anesthetics have been shown to affect the learning and memory functions of rodents many days after the exposure [3], [4], indicating that volatile anesthetics may play a role in the development of postoperative cognitive decline (POCD), a recognized clinical entity that has drawn significant attention from the scientific community and the public [5].

POCD refers to cognitive decline as reflected by poorer performance in neuropsychological tests after surgery than that before surgery. Patients with POCD may have an increased mortality [6], [7] and a higher chance of leaving the labor market prematurely [6]. Although POCD resolves in days or weeks in most patients, it still exists in ∼10% elderly patients (≥60 years) at 3 months after a non-cardiac surgery [7], [8]. Age is a risk factor for this middle-term POCD [7], [8]. However, young adults are as likely as elderly to suffer short-term POCD but may not develop middle-term POCD [7].

It is well-known that neuroinflammation impairs cognitive functions [9], [10]. A recent study showed that neuroinflammation is critical for the development of cognitive impairment in young adult mice after an open tibial fracture fixation under general anesthesia [11]. However, the mechanisms for general anesthetics-induced cognitive impairment are not known. We and others have shown that isoflurane increases cytokine production in the rat or mouse brains [3], [12]. Thus, we designed this study to determine whether proinflammatory cytokines, such as interleukin 1β (IL-1β), play an important role in isoflurane-induced cognitive impairment. Also, we determined which processes of the learning and memory functions are affected by isoflurane. To simulate clinical situation, 18-month old rats that are in their early elderly adulthood were used in the study. In addition, young adult wild-type and IL-1β deficient mice were used to facilitate mechanistic study because young adult patients also can suffer from POCD and 18-month old IL-1β deficient mice are not immediately available.

## Results

### Isoflurane Exposure Impaired the Cognitive Functions of 18-month Old Rats

**Figure 1:**
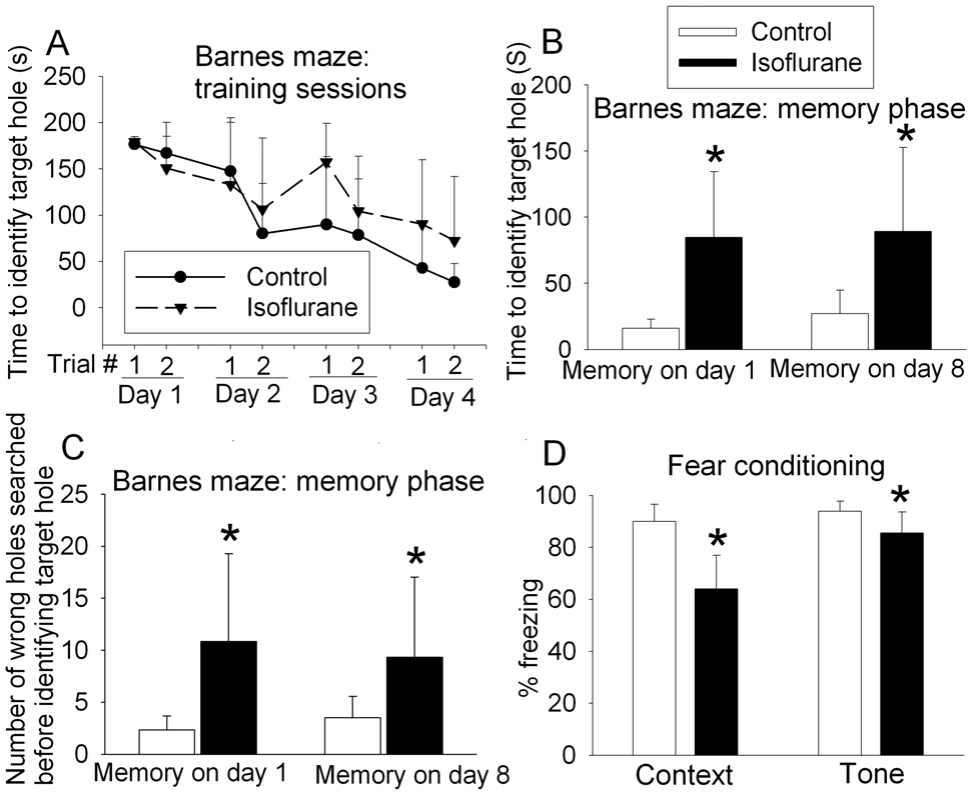
Isoflurane-induced cognitive impairment measured by Barnes maze and fear conditioning. Eighteen-month-old Fisher 344 rats were exposed to or were not exposed to 1.2% isoflurane for 2 h. They were subjected to Barnes maze 2 weeks later or fear conditioning 27 days later. A: performance in the training sessions of Barnes maze test. There is a significant effect of training sessions on the latency to identify the target hole (P<0.001 by two-way repeated measures analysis of variance). B: latency to identify the target hole at 1 day or 8 days after the training sessions. C: number of other hole searched before identifying the target hole at 1 day or 8 days after the training sessions. D: performance in fear conditioning test. Results are mean±S.D. (n = 6). *P<0.05 compared with the corresponding control in panels B, C and D by t-test.

There was no episode of hypoxia [defined as pulse oximeter oxygen saturation (SpO_2_) <90%] during isoflurane exposure and the blood pressures of rats during isoflurane anesthesia were similar to those of control rats as we reported before [13]. The time for 18-month old rats in control and isoflurane-exposed groups to find the target hole was decreased with increased training in the Barnes maze (Fig. 1A). The effects of training sessions on the latency for rats to find the target hole were very significant (P<0.001), suggesting that control and isoflurane-exposed rats are able to acquire and recall the learnt information. The effects of isoflurane on the latency for rats to find the target hole during the training sessions did not reach statistical significance (P = 0.111). However, the latency for isoflurane-exposed rats to identify the target hole was significantly longer than that by control rats at 1 day or 8 days after the training sessions (Fig. 1B). Consistent with this result, isoflurane-exposed animals searched more non-target holes than control rats before they identified the target hole (Fig. 1C). Isoflurane-exposed rats also had significantly less freezing behavior than control animals in the context- and tone-related fear conditioning tests (Fig. 1D). These results suggest that isoflurane-exposed rats had significant cognitive impairment.

### Isoflurane Exposure Impaired the Learning, but did not Affect the Recall, of 18-month Old Rats

**Figure 2:**
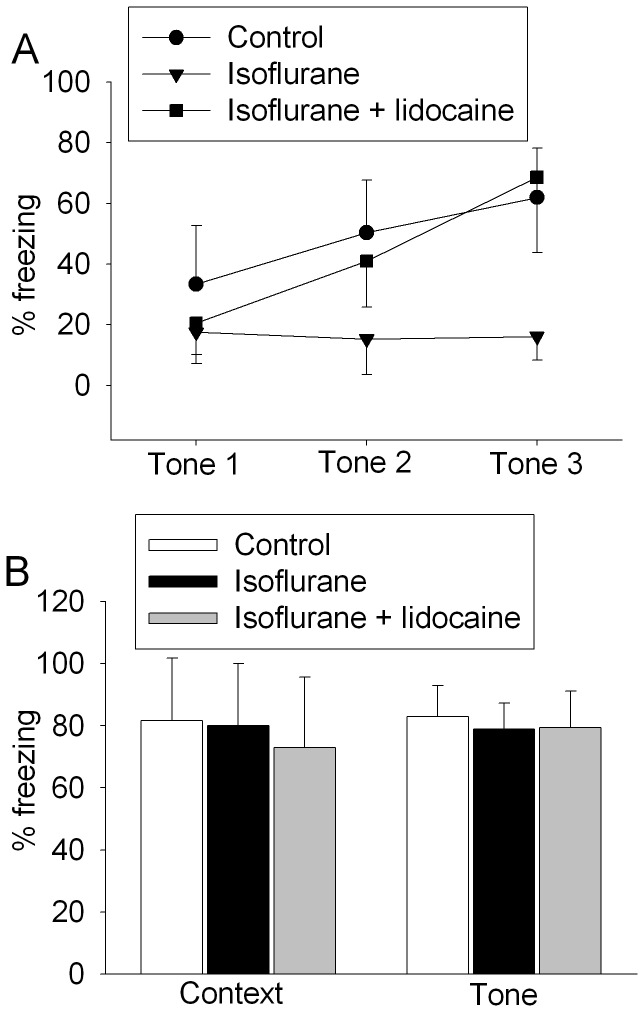
Isoflurane-induced learning impairment measured by fear conditioning test. A: freezing behavior during training sessions for 18-month old Fisher 344 rats that were exposed to isoflurane in the presence or absence of lidocaine 27 days ago. Results are means±S.D. (n = 6 for control and isoflurane only groups and  = 7 for isoflurane plus lidocaine group). There is a significant effect of training trial (P<0.001, among the control animals and animals exposed to isoflurane plus lidocaine), isoflurane use (P = 0.002, control animals vs. animals exposed to isoflurane only) and lidocaine use (P = 0.003, animals exposed to isoflurane only vs. animals exposed to isoflurane plus lidocaine) on the freezing behavior. Statistical analysis was performed by two-way analysis of variance. B: freezing behavior during the context- and tone-related fear conditioning tests for 18-month old Fisher 344 rats that first had the training sessions and were exposed to isoflurane in the presence or absence of lidocaine at 30 min after the training sessions. Results are means±S.D. (n = 4 for control group and  = 5 for the isoflurane only and isoflurane plus lidocaine groups).

To determine which process of the learning and memory functions has been affected by isoflurane, we determined the freezing behavior in the interval (1 min) after each of the three tone-foot shock pairs (training trials) during the fear conditioning phase. Rats had more freezing behavior with more training trials (Fig. 2A). This effect was very significant (P<0.001). Isoflurane exposure before the training trials significantly reduced this learning process (P = 0.002 for this effect). The use of lidocaine, a local anesthetic with anti-inflammatory effect [14], during isoflurane exposure has been shown to attenuate isoflurane-induced cognitive impairment in our previous study [13]. This use of lidocaine also reversed the isoflurane-reduced freezing behavior during the training trials (P = 0.003 for this lidocaine effect) (Fig. 2A). These results suggest that isoflurane impairs the learning process and that lidocaine attenuates this isoflurane effect.

To determine whether isoflurane affects the processes after learning/acquisition, rats first were subjected to the three tone-foot shock pairs. Thirty minutes later, they were exposed to isoflurane and the context- and tone-related fear conditioning responses were measured 48 h after isoflurane exposure. There was no difference in the freezing behavior among the control, isoflurane-exposed and isoflurane plus lidocaine-exposed animals during the context- and tone-related fear conditioning tests (Fig. 2B). These results suggest that isoflurane may not affect the recall of learnt information in these animals.

### Isoflurane Exposure Increased the Expression of IL-1β, IL-6 and Activated Caspase 3 in the Hippocampus of 18-month Old Rats

**Figure 3:**
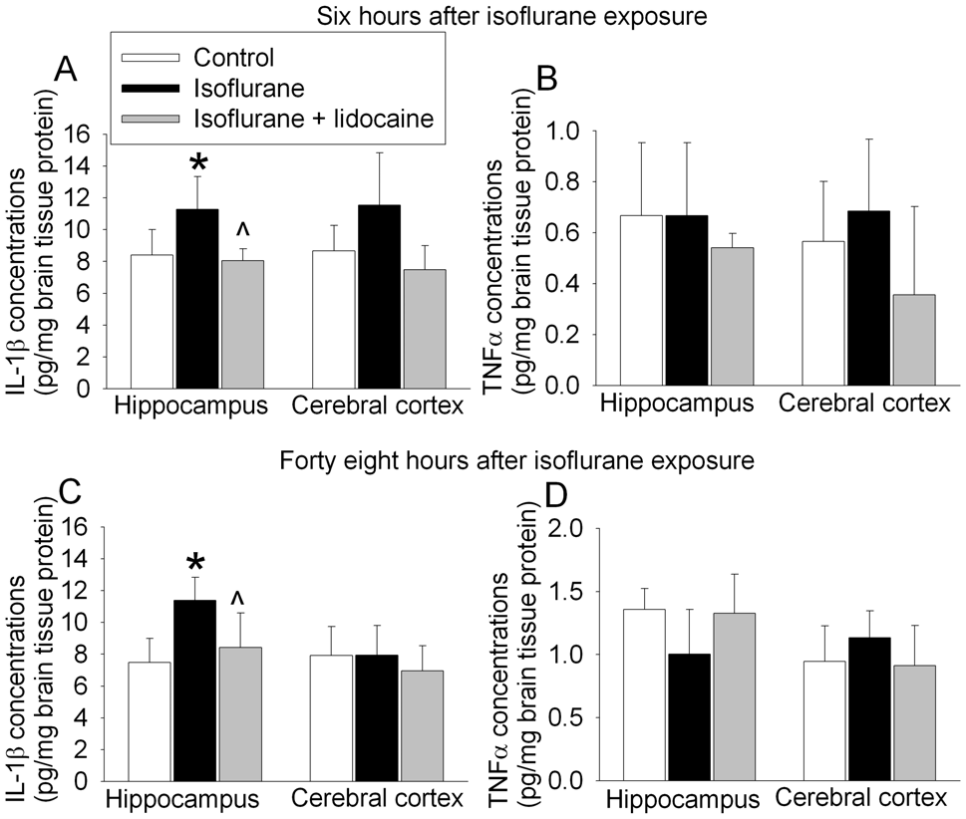
Isoflurane effects on interleukin 1β (IL-1β) and tumor necrosis factor α (TNFα) contents in rat brain tissues. A and B: eighteen-month-old Fisher 344 rats were exposed to or were not exposed to 1.2% isoflurane in the presence or absence of lidocaine for 2 h. Hippocampus and cerebral cortex were harvested at 6 h after anesthetic exposure for ELISA of IL-1β or TNFα content. C and D: eighteen-month-old Fisher 344 rats had training sessions of fear conditioning test and 30 min later were exposed to or were not exposed to 1.2% isoflurane in the presence or absence of lidocaine for 2 h. Hippocampus and cerebral cortex were harvested at 48 h after anesthetic exposure for ELISA of IL-1β or TNFα content. Results are means±S.D. (n = 4). *P<0.05 compared to control. ? P<0.05 compared to isoflurane alone. Statistical analysis was performed by one-way analysis of variance.

**Figure 4:**
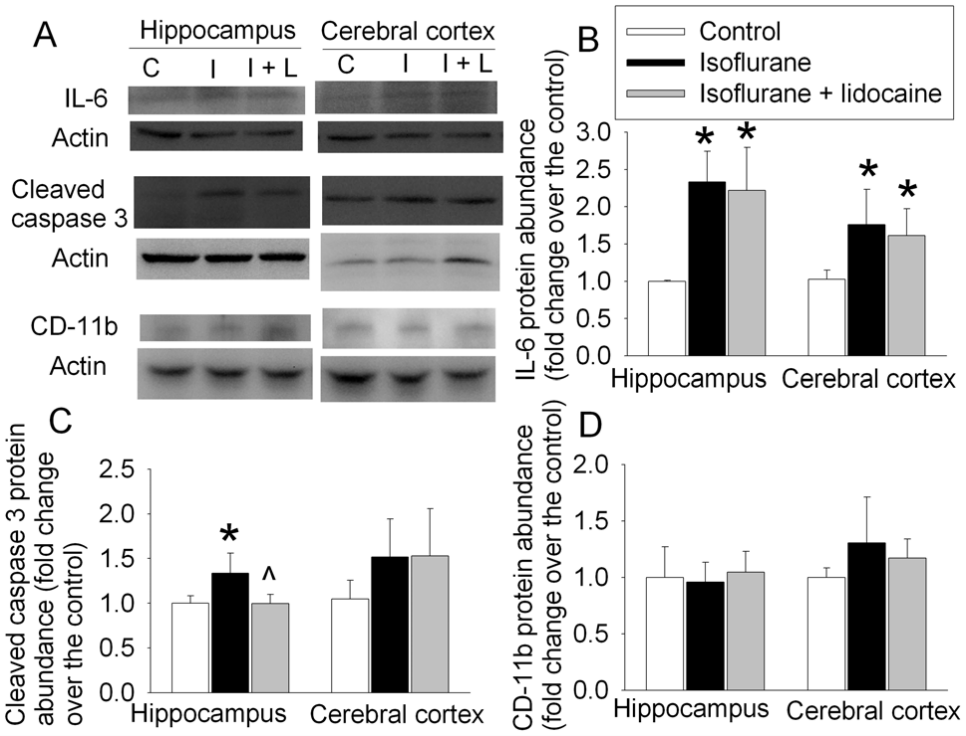
Isoflurane effects on the expression of interleukin 6 (IL-6), activated/cleaved caspase 3 and cluster of differentiation 11b (CD-11b) in rat brain tissues. Eighteen-month-old Fisher 344 rats were exposed to or were not exposed to 1.2% isoflurane in the presence or absence of lidocaine for 2 h. Hippocampus and cerebral cortex were harvested at 6 h after anesthetic exposure for Western blotting. A: representative Western blot images. B, C and D: graphic presentation of the IL-6, cleaved caspase 3 and CD-11b protein abundance quantified by integrating the volume of autoradiograms from 4 rats for each experimental condition. Values in graphs are expressed as fold changes over the mean values of control animals and are presented as the means±S.D. *P<0.05 compared with the control group. ? P<0.05 compared to isoflurane alone. Statistical analysis was performed by one-way analysis of variance. C: control, I: isoflurane, I+L: isoflurane plus lidocaine.

Animals exposed to isoflurane had increased IL-1β and activated caspase 3 expression in the hippocampus at 6 h after the exposure. This increase was attenuated by lidocaine. There was no significant difference in tumor necrosis factor α (TNFα) content in the hippocampus and cerebral cortex among three groups of rats (Figs. 3A & B). IL-6 in the hippocampus and cerebral cortex at 6 h after isoflurane exposure was significantly increased and this increase was not affected by lidocaine. The expression of cluster of differentiation 11b (CD-11b), a marker of microglial activation [11], was not changed by isoflurane and lidocaine (Fig. 4).

**Figure 5:**
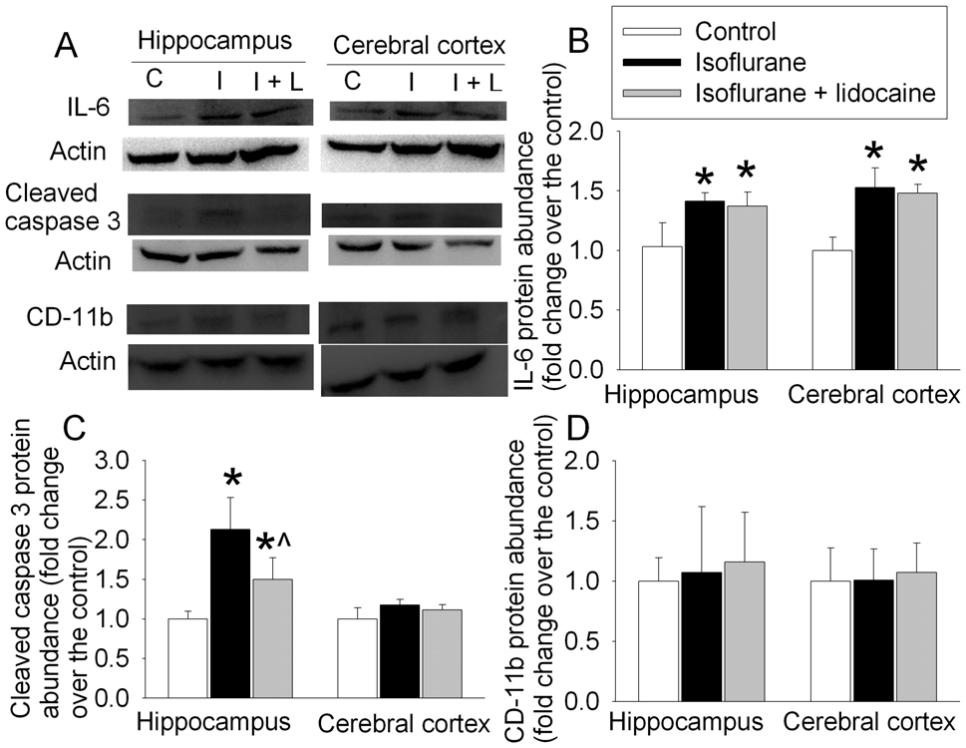
Isoflurane effects on the expression of interleukin 6 (IL-6), activated/cleaved caspase 3 and cluster of differentiation 11b (CD-11b) in rat brain tissues. Eighteen-month-old Fisher 344 rats had the training sessions of fear conditioning and 30 min later were exposed to or were not exposed to 1.2% isoflurane in the presence or absence of lidocaine for 2 h. Hippocampus and cerebral cortex were harvested at 48 h after anesthetic exposure for Western blotting. A: representative Western blot images. B, C and D: graphic presentation of the IL-6, cleaved caspase 3 and CD-11b protein abundance quantified by integrating the volume of autoradiograms from 4 rats for each experimental condition. Values in graphs are expressed as fold changes over the mean values of control animals and are presented as the means±S.D. *P<0.05 compared with the control group. ? P<0.05 compared to isoflurane alone. Statistical analysis was performed by one-way analysis of variance. C: control, I: isoflurane, I+L: isoflurane plus lidocaine.

Similar to the change patterns at 6 h after isoflurane exposure, there was a significant increase in IL-1β and activated caspase 3 in the hippocampus of rats at 48 h after the fear conditioning training trials and then isoflurane exposure. This increase was attenuated by lidocaine (Figs. 3C and 5). The expression of IL-6 in the hippocampus and cerebral cortex at 48 h after fear conditioning trials and then isoflurane exposure was increased and this increase was not affected by lidocaine. The expression of TNFα and CD-11b at this time-point also was not affected by isoflurane and lidocaine exposure (Figs. 3D and 5).

**Figure 6:**
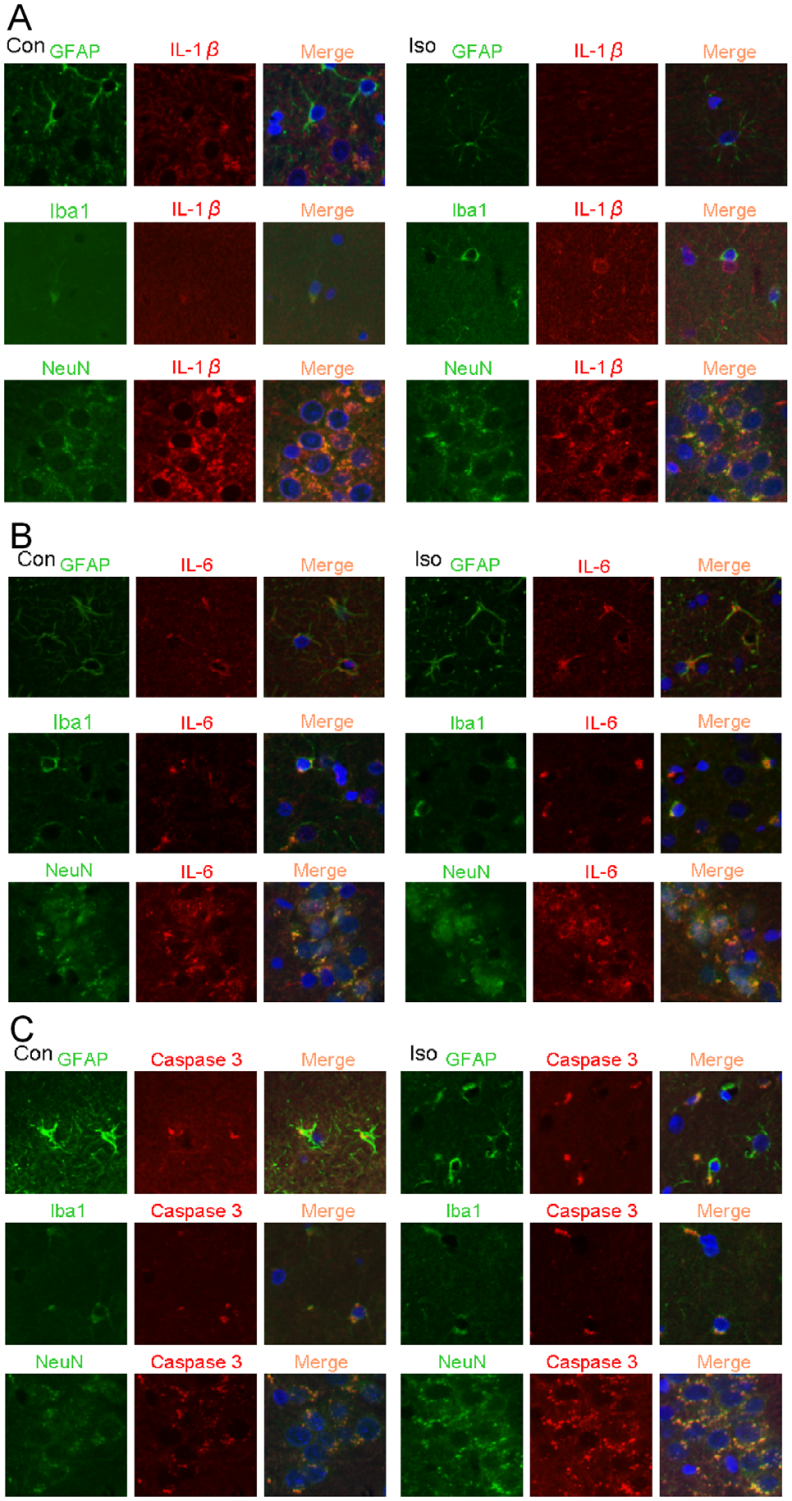
Expression of interleukin 1β (IL-1β), IL-6 and activated/cleaved caspase 3 in rat brain tissues. Eighteen-month-old Fisher 344 rats were exposed to or were not exposed to 1.2% isoflurane for 2 h. Hippocampus was harvested at 48 h after isoflurane exposure for immunofluorescent staining of IL-1β (red), IL-6 (red), cleaved caspase 3 (red), NeuN (green), glial fibrillary acidic protein (GFAP, green) and ionized calcium binding adaptor molecule 1 (Iba1, green). The merged panels also include Hoechst staining (blue) to show cell nuclei. A: co-staining of IL-1β with GFAP, Iba1 and NeuN. B: co-staining of IL-6 with GFAP, Iba1 and NeuN. C: co-staining of cleaved caspase 3 with GFAP, Iba1 and NeuN. Con: control, Iso: isoflurane.

IL-1β positive staining was co-localized with the positive staining for NeuN, a neuronal marker, but did not appear to be co-localized with positive staining of glial fibrillary acidic protein (GFAP), an astrocytic marker, or ionized calcium binding adaptor molecule 1 (Iba1), a microglial marker, in the hippocampus (Fig. 6A). On the other hand, positive staining for IL-6 or active caspase 3 was co-localized with NeuN, GFAP and Iba1 (Figs. 6B and 6C). The density of Iba1 in the dentate gyrus of control and isoflurane-exposed rats was 23±3 and 25±2 (arbitrary unit, n = 4, P = 0.482 by t-test). Isoflurane exposure did not significantly affect the expression of neuron-specific proteins of 18-month old rats.

**Figure 7:**
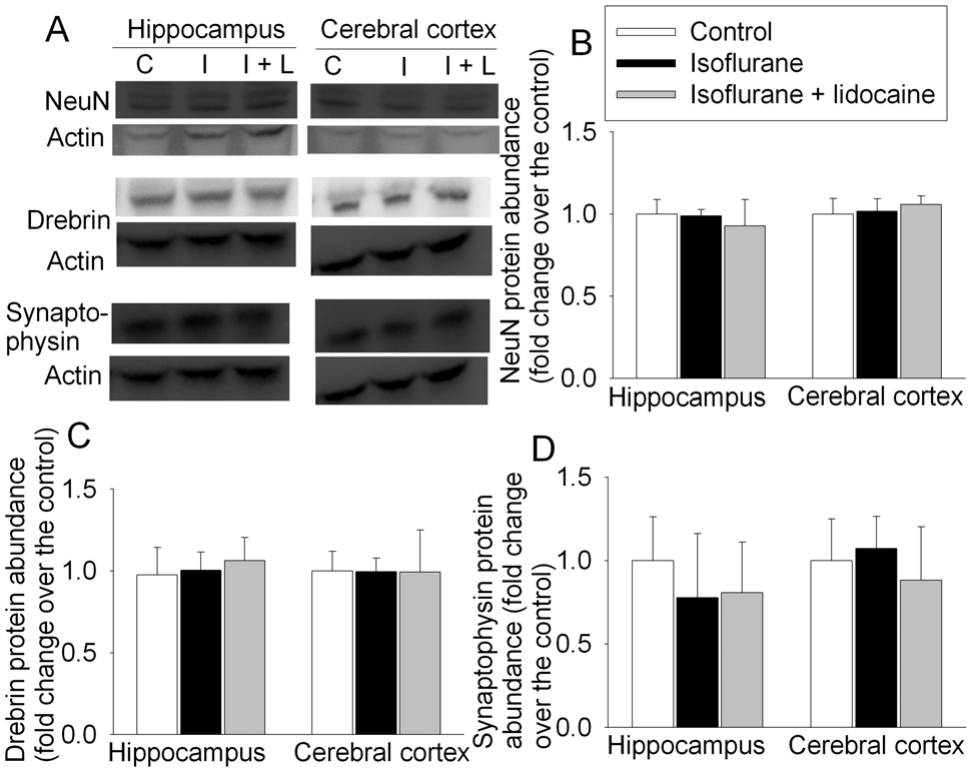
Isoflurane effects on the expression of NeuN, drebrin and synaptophysin in rat brain tissues. Eighteen-month-old Fisher 344 rats had the training sessions of fear conditioning and 30 min later were exposed to or were not exposed to 1.2% isoflurane in the presence or absence of lidocaine for 2 h. Hippocampus and cerebral cortex were harvested at 48 h after anesthetic exposure for Western blotting. A: representative Western blot images. B, C and D: graphic presentation of the NeuN, drebrin and synaptophysin protein abundance quantified by integrating the volume of autoradiograms from 4 rats for each experimental condition. Values in graphs are expressed as fold changes over the mean values of control animals and are presented as the means±S.D. C: control, I: isoflurane, I+L: isoflurane plus lidocaine.

There was no difference in the expression of NeuN (a neuron-specific protein), drebrin (a dendritic spine protein) and synaptophysin (a synaptic protein) in the hippocampus and cerebral cortex among the control rats or rats that had fear conditioning training trials and then exposure to isoflurane in the presence or absence of lidocaine 48 h before the tissues were harvested for Western blotting (Fig. 7).

### Isoflurane Exposure Increased the IL-1β Expression and Impaired the Cognitive Functions of Wild-type Mice but did not Affect the Cognitive Functions of IL-1β Deficient Mice

Unlike rats that were intubated and mechanically ventilated to maintain normal end tidal CO_2_ during isoflurane exposure, mice were spontaneously breathing during the exposure. One concern is that isoflurane can cause significant respiratory inhibition that leads to CO_2_ accumulation in these mice. At the end of the 2-h isoflurane exposure, blood samples taken from the left heart of the mice showed that pH, PCO_2_ and PO_2_ were 7.325±0.037, PCO_2_ 46. 5±0.9 mmHg and PO_2_ 224±49 mmHg (n = 4), respectively. These pH and PCO_2_ values are almost identical to those of normal young adult mice or young rats without general anesthesia [15], [16].

**Figure 8:**
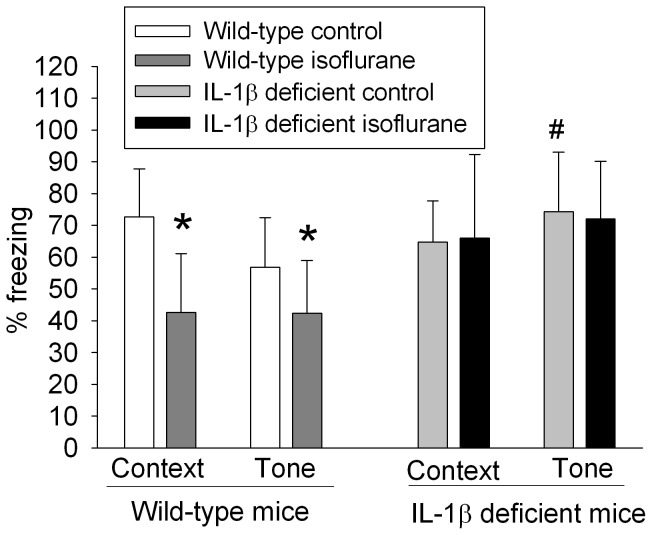
Isoflurane effects on cognitive functions assessed by fear conditioning. Ten-week old male C57BL/6J (wild-type) mice or interleukin 1β (IL-1β) deficient mice were exposed to 1.4% isoflurane for 2 h and then subjected to the fear conditioning test 48 h later. Results are means±S.D. (n = 15 for the wild-type mice and 6 for the IL-1β deficient mice). *P<0.05 compared with the corresponding control group. # P<0.05 compared to the wild-type control mice. Statistical analysis was performed by t-test.

Isoflurane significantly increased IL-1β in the hippocampus of young adult mice at 6 h after the exposure (4.6±0.3 and 5.1±0.6 pg/mg brain tissue proteins for control and isoflurane-exposed mice, respectively, n = 8 for control and 7 for isoflurane-exposed mice, P = 0.048 by t-test). Mice exposed to isoflurane at 48 h before the fear conditioning training trials had less freezing behavior in the context- and tone-related fear conditioning tests. This isoflurane effect was not present in the IL-1β deficient mice (Fig. 8), suggesting a role of IL-1β in this isoflurane effect.

## Discussion

We showed here that 18-month old rats that are considered to be in their early elderly stage or late phase of middle age had poorer cognitive performance as assessed by Barnes maze and fear conditioning tests if they had a prior exposure to 1.2% isoflurane for 2 h. One minimum alveolar concentration (the concentration at which 50% of animals do not have a motor response to painful stimuli) of isoflurane in rats is ∼1.5% [17]. Many surgeries often require more than 2 h to complete. Thus, our results suggest that isoflurane used in a clinically relevant manner impairs the cognitive functions of rats. This finding is consistent with our previous report and reports from others [3], [4], [13]. A novel finding of our study is that isoflurane may impair the learning/acquisition but may not affect the recall of learnt information. Learning and memory functions require various processes including acquisition, consolidation and recall. Our results showed that prior exposure to isoflurane reduced the freezing behavior of rats during the training sessions of fear conditioning. This isoflurane effect was blocked by lidocaine, a local anesthetic that attenuated isoflurane-induced cognitive functions of rats in our previous study [13]. On the other hand, there was no difference in freezing behavior in the context- and tone-related fear conditioning if they first had training sessions and then were exposed to isoflurane, isoflurane plus lidocaine or oxygen only. These results suggest that isoflurane does not affect the recall of information that is acquired.

One important and novel finding of our study is that IL-1β may play a critical role in isoflurane-induced cognitive impairment. Volatile anesthetics have been shown to impair cognitive functions of animals [3], [4], [13], although no effect or improvement of cognitive functions also has been reported for these anesthetics in animal studies, especially when using young adult animals [4], [17], [18], [19]. Increased caspase 3 activation and amyloid β production in the brain have been observed in acute phase after volatile anesthetic exposure [20]. However, it is not known whether these brain changes contribute to the cognitive dysfunction after anesthetic exposure. We have shown that amyloid β content in rat brain is not increased when they have significant cognitive impairment after isoflurane exposure [13], suggesting that amyloid β may not play an important role in the cognitive dysfunction. We showed here that isoflurane increased IL-1β and that lidocaine attenuated this increase and the isoflurane-induced cognitive impairment in the 18-month old rats. Similar to a previous study [21], isoflurane also impaired the cognitive functions of wild-type young adult mice in our study. In addition, isoflurane increased IL-1β in their hippocampi. However, isoflurane did not affect the cognitive functions of IL-1β deficient mice. These results strongly suggest the critical role of IL-1β in isoflurane-induced cognitive impairment. Consistent with our findings, a recent study shows that cognitive impairment after an open tibial fracture fixation under general anesthesia in young adult mice is mediated by IL-1β [11]. However, the study did not show that general anesthesia alone (2.1% isoflurane for 20 min plus 0.1 mg/kg buprenorphine) affected cognitive functions of the young adult mice, which is different from our results and the results of a previous study [21]. Also, the same study did not show anesthesia alone increase IL-1β in the mouse brain. A recent study showed that 2.1% isoflurane for 15 min plus 0.1 mg/kg buprenorphine did not affect hippocampus-dependent memory but impaired remote neocortex-dependent memory of young adult mice [22]. The reasons for the different findings among the studies are not known. However, the previous study that failed to show anesthetic effects on cognition and IL-1β levels in the brain used a much shorter isoflurane exposure than ours. The short exposure may not be long enough to induce those brain changes. Also, different learning and memory paradigms used to test animals may contribute to the different findings in the studies.

In addition to IL-1β, isoflurane also increased IL-6 in the brain. IL-6 is a proinflammatory as well as anti-inflammatory cytokine [23], [24]. Increased IL-6 is associated with decreased cognitive performance in humans [25], [26]. It is conceivable that IL-6 plays a role in the isoflurane-induced cognitive dysfunction in our rats. However, lidocaine attenuated isoflurane-induced cognitive impairment but did not change the isoflurane-induced increase of IL-6. Also, isoflurane did not induce impairment in learning and memory in the IL-1β deficient mice. These results suggest that IL-6 may not play a major role in isoflurane-induced cognitive impairment. In this study, we did not observe a change in TNFα, a proinflammatory cytokine [10], at 6 and 48 h after isoflurane exposure. Measuring TNFα at other time-points may be needed to determine the effects of isoflurane on TNFα expression and the role of TNFα in the isoflurane-induced cognitive impairment.

IL-1β is a proinflammatory cytokine [11]. Its increase after isoflurane exposure in the brain suggests neuroinflammation. However, the expression of CD-11b and Iba1, indicators of reactive microglia [11], was not changed significantly after isoflurane exposure. Consistent with our finding, a previous study showed that CD-11b expression in mouse brain was not changed by isoflurane as assessed by an immunohistochemistry method [11]. These findings suggest that a small increase of proinflammatory cytokines after isoflurane exposure may not cause a picture of significant neuroinflammation as assessed by CD-11b or Iba1 expression.

We observed a significant increase of cleaved/activated caspase 3 in the hippocampi of rats after isoflurane exposure. This increase was attenuated by lidocaine. Caspase 3 activation may be a biomarker for apoptosis [27], [28]. Our results do not suggest that isoflurane causes significant neuronal death because the expression of neuN, drebrin and synaptophysin was not changed significantly at 48 h after isoflurane exposure. Also, our previous study showed that isoflurane did not change the neuronal density in the hippocampus and the synaptic density in the hippocampal stratum radiatum of 18-month old rats at 29 days after the exposure [13]. Together, these results suggest that isoflurane exposure may not significantly change the neural structures in the hippocampus.

Our immunohistochemistry results suggest that IL-1β may be from neurons and that IL-6 and activated caspase 3 are expressed in neurons, astrocytes and microglia of control rat hippocampus. This cell type-specific distribution of cytokines was not affected by isoflurane. Consistent with our results, TNFα in mouse cerebral cortex was found mainly in neurons but not in astrocytes [12].

Our previous study showed that a 2-h isoflurane exposure impaired cognitive functions in aged rats and that lidocaine attenuated this impairment. We then showed that significant biochemical or structural changes in the brain were not observed at 29 days after the isoflurane exposure [13]. In this study, we presented data that isoflurane caused proinflammatory cytokine increase in the brain. With the use of lidocaine as a tool, our data also indicate that isoflurane-induced cognitive impairment may be mainly on the learning process under current experimental conditions and that this impairment may be mediated by IL-1β. These novel findings provide evidence to support the hypothesis that mild neuroinflammation contributes to isoflurane-induced cognitive impairment.

Our current study showed that lidocaine attenuated isoflurane-induced cognitive impairment and increase of hippocampal IL-1β and activated caspase 3 levels. Lidocaine may inhibit the nuclear factor κB signaling to reduce proinflammatory cytokine production [29]. Our results clearly suggest the effects of lidocaine on the brain. Lidocaine can use transport system to penetrate the blood-brain barrier to get into the brain [30]. Also, penetration of peripheral macrophages into the brain can induce IL-1β production in the brain [31]. Since lidocaine has the ability of inhibiting mobility of inflammatory cells including macrophages [32], it is possible that lidocaine via its effects on the peripheral tissues/cells induces the effects on the brain as observed in this study. Lidocaine has a relatively short elimination half-life (∼90 min). Here, we observed cognitive improvement in rats many weeks after the isoflurane and lidocaine application compared with rats exposed to isoflurane only. This phenomenon may be due to the inhibition of the detrimental effects of isoflurane in the early phase so that subsequent events leading to the delayed cognitive dysfunction will not occur. The active lidocaine metabolites, such as monoethylglycinexylidide, that have longer half-life than lidocaine may also contribute to this phenomenon.

We observed that the IL-1β deficient mice had better tone-related learning and memory than wild-type mice under control condition. This finding is consistent with our proposal that IL-1β increase is a critical step for isoflurane-induced cognitive impairment. Similarly, IL-1β is considered to be a mediator for age-dependent cognitive impairment [33].

We showed here that isoflurane induced cognitive impairment in rats and mice. One has to be very cautious to extrapolate these findings to humans because significant species difference in response to isoflurane may exist. Also, most patients receive anesthesia because of surgery, although some patients may receive general anesthesia without having surgery (for example, radiology image studies under general anesthesia). Anesthetic effects may be different in cells or organisms in the presence or absence of stress stimuli, such as surgery. Further studies are needed to determine the interaction between anesthetics/anesthesia and surgery and its contribution to the development of POCD.

In summary, our study showed that isoflurane impaired the acquisition/learning of aged rats but did not affect their recall of learnt information. This isoflurane effect may be mediated by IL-1β. Lidocaine may attenuate the long-term cognitive impairment of aged rats after isoflurane exposure by inhibiting the early phase of mild brain inflammation.

## Materials and Methods

The animal protocol was approved by the institutional Animal Care and Use Committee of the University of Virginia (Charlottesville, VA; Protocol number 3114). All animal experiments were carried out in accordance with the National Institutes of Health Guide for the Care and Use of Laboratory Animals (NIH publications number 80–23) revised in 1996.

### Animals

Eighteen-month-old male Fisher 344 rats weighing 470–550 g were from the National Institutes of Health (Bethesda, MD). Ten-week old male C57BL/6J mice (stock number 000664) and IL-1β deficient mice (stock number 005576) were obtained from the Jackson Laboratories (Bar Harbor, ME). These IL-1β deficient mice had a gene background similar to that of C57BL/6J mice.

### Isoflurane Exposure

Rats were exposed to isoflurane as we described before [34]. Briefly, anesthesia was induced by placing rats in a chamber gassed with 3% isoflurane in oxygen. They then were intubated with a 14-gauge catheter and mechanically ventilated with isoflurane carried by 100% O_2_ to maintain end tidal isoflurane concentration at 1.2%. The inhaled and exhaled gas concentrations were monitored continuously with a DatexTM infrared analyzer (Capnomac, Helsinki, Finland). The ventilator settings usually were as follows: 2 ml as the tidal volume and respiratory rate at 60 breaths/min. The settings were adjusted to maintain the end tidal CO_2_ at ∼32 mm Hg. Rectal temperature was maintained at 37°C ±0.5°C. Heart rate and SpO_2_ were measured continuously during anesthesia with a MouseOx™ Pulse Oximeter (Harvard Apparatus, Holliston, MA). Non-invasive blood pressure was measured by using a CODA Monitor (Kent Scientific Corp., Torrington, CT). After a 2-h isoflurane exposure, isoflurane application was stoped. Rats were extubated when responsive. They were recovered for 20 min in a chamber gassed with 100% O_2_ and at 37°C, and then were placed in their home cage.

Mice were exposed to isoflurane by placing them in a chamber that was gassed with 1.4% isoflurane in oxygen for 2 h. Part of the chamber was submerged in a water-bath at 37°C. Animals also were monitored by a MouseOx™ Pulse Oximeter to make sure their SpO_2_ was >96% during isoflurane exposure. At the end of isoflurane exposure, 4 animals were used to collect blood from the left heart for blood gas analysis. The other animals were placed back to their home cage.

Since isoflurane was carried by 100% O_2_, rats or mice in the control group were kept in a chamber that was gassed with 100% O_2_ for 2 h.

### Lidocaine Application to Rats

Animals in the isoflurane plus lidocaine group received lidocaine via a tail vein. As we did before [13], lidocaine was given intravenously at 1.5 mg/kg as a bolus and then 2 mg/kg/h during the 2-h isoflurane exposure. Lidocaine was dissolved in normal saline at 8 mg/ml. The rats in the isoflurane only group were given the same volume of saline.

### Barnes Maze

Two weeks after isoflurane exposure, animals were subjected to Barnes maze in the same way as we described before [13] to test their spatial learning and memory. Barnes maze is a circular platform with 20 equally spaced holes (SD Instruments, San Diego, CA). One hole was connected to a dark chamber that was called target box. Each time, animals were placed in the middle of the platform and were encouraged to find the target box by aversive noise (85 dB) and bright light (200 W) shed on the platform. They went through a spatial acquisition phase that consisted of training on 4 days with 2 trials per day, 3 min per trial and 15 min between each trial. The reference memory of the animals was tested on day 5 (short-term retention) and day 12 (long-term retention). One trial on each of these two days was performed. The rats were not subjected to any tests during the period from day 5 to day 12. The latency and number of errors to find the target box during each trial were recorded with the assistance of ANY-Maze video tracking system (SD Instruments).

### Fear Conditioning

Rats or mice were subjected to fear conditioning test at various times in relation to isoflurane exposure to test their non-effort-dependent learning and memory [35]. As we described before [13], each animal was placed in a test chamber wiped with 70% alcohol and subjected to 3 tone-foot shock pairings (tone: 2000 Hz, 85 db, 30 s; foot shock: 1 mA, 2 s) with an intertrial interval 1 min in the chamber placed in a relatively dark room (training sessions). The animal returned to home cage 30 s after the conditioning training and was placed back to the chamber 24 h later for 8 min (rats) or 6 min (mice) in the absence of tone and shock. The amount of time with freezing behavior was recorded in an 8 s (rats) or 6 s (mice) interval. The animal was placed 2 h later in a test chamber that was different in context and smell from the first test chamber and was in a relatively light room. Freezing behavior was recorded for 3 min without the auditory conditioning stimulus. The auditory stimulus then was turned on for 3 cycles, each cycle for 30 s followed by 1-min inter-cycle interval (4.5 min in total). The freezing behavior in this 4.5 min was recorded. Freezing behavior recorded in the video was evaluated by an observer who was blind to group assignment. These sets of experiments test hippocampus-dependent (context-related) and hippocampus-independent (tone-related) learning and memory functions [35].

### Brain Tissue Harvest

Six hours after isoflurane exposure, rats were deeply anesthetized with isoflurane and perfused transcardially with saline. The right hippocampi and cortex were dissected out immediately for the Western blotting of various proteins. The left hippocampi and cortex were taken out for enzyme-linked immunosorbent assay (ELISA) of IL-1β and TNFα.

Similarly, 6 h after isoflurane exposure, hippocampi of wild-type mice were harvested for ELISA of IL-1β.

In the third experiment, rats first had the training sessions of the fear conditioning test. Thirty minutes later, they were subjected to isoflurane exposure. Their hippocampi and cerebral cortices were harvested 48 h after isoflurane exposure for ELISA of IL-1β and TNFα, and Western blotting of various proteins.

### Western Blotting

Brain tissues were homogenized in RIPA buffer (catalogue number: 89900; Thermo Scientific, Worcester, MA) containing protease inhibitor cocktail (catalogue number: P2714; Sigma, St Louis, MO) and protease inhibitor mixture (catalogue number: 1697498; Roche Applied Science, Indianapolis, IN). Homogenates were centrifuged at 13,000 g at 4°C for 20 min. The supernatant was saved and its protein concentration was determined by the Bradford assay.

Fifty microgram proteins per lane were separated on a polyacrylamide gel and then blotted onto a polyvinylidene difluoride membrane. The membranes were blocked with Protein-Free T20 Blocking Buffer (catalogue number: 37573, Lot LB141635; Thermo Scientific, Waltham, MA) and incubated with the following primary antibodies: rabbit polyclonal anti-synaptophysin antibody (1∶1000; catalogue number: 4329; Cell Signaling Technology, Inc., Danvers, MA), mouse monoclonal anti-drebrin antibody (1∶1000; catalogue number: ab12350; Abcam, Cambridge, MA), mouse monoclonal anti-NeuN, clone A06 antibody (1∶500; catalogue number: MAB377; Millipore Cor. Billerica, MA), rabbit polyclonal anti-cleaved caspase-3 antibody (1∶1000; catalogue number: 9664; Cell Signaling Technology, Inc.), rabbit polyclonal anti-CD-11b (1∶500; catalogue number: ab75476; Abcam), rabbit polyclonal anti-IL-6 (1∶500; catalogue number: ab6672; Abcam) and rabbit polyclonal anti-β-actin polyclonal antibody (1∶2000; catalogue number: A2228; Sigma). Appropriate secondary antibodies were used. Protein bands were visualized using a Genomic and Proteomic Gel Documentation (Gel Doc) Systems from Syngene (Frederick, MD). The protein band intensities of NeuN, cleaved-caspase 3, CD-11b, IL-6, synaptophysin and drebrin were normalized by the corresponding band intensities of β-actin from the same samples to reduce loading errors. The results from animals under various experimental conditions then were normalized by mean values of the corresponding control animals.

### Quantification of IL-1β and TNFα

Brain tissues were homogenized on ice in 20 mM Tris–HCl buffer (pH = 7.3) containing protease inhibitors (10 µg/ml aproteinin, 5 µg/ml peptastin, 5 µg/ml leupeptin, and 1 mM phenylmethanesulfonylfluoride). Homogenates were centrifuged at 13,000 g for 20 min at 4°C. The supernatant was saved and Bradford protein assay of the supernatant was performed for each sample. ELISA kits for measuring rat IL-1β and TNFα (catalogue number: DY501 and RTA00, respectively; R&D Systems, Minneapolis, MN) were used to quantify the contents of these cytokines in the samples according to the manufacturer’ instructions. The quantity of IL-1β and TNFα in each brain sample was standardized to the protein contents.

### Immunohistochemistry

The tissue preparation of immunofluorescent staining of brain sections were performed as we described previously [36]. Briefly, 48-h after isoflurane exposure, 18-month old rats were deeply anesthetized with isoflurane and perfused transcardially with saline and then 4% phosphate-buffered paraformaldehyde. Brains were harvested and stored in the same fixative solution for 24 h. Five-micrometer-thick paraffin coronal sections at bregma −3.80 mm were cut by a microtome. Antigen retrieval was performed with a microwave heating for 20 min in a Tris base/ethylenediaminetetraacetic acid (Tris/EDTA) buffer (pH = 9.0) containing 0.05% tween-20. Sections were washed in Tris-buffered saline (TBS) plus 0.025% triton-X 100. They were then blocked in 10% donkey serum with 1% bovine serum albumin in TBS for 2 h at room temperature. The primary antibodies used were: rabbit polyclonal anti-IL-6 antibody (1∶200; catalog number: ab6672; Abcam), rabbit anti-cleaved caspase-3 antibody (1∶400; catalog number: 9664; Cell Signaling Technology), rabbit polyclonal anti-IL-1β antibody (1∶50; catalog number: sc-7884; Santa Cruz Biotechnology), mouse monoclonal anti-GFAP antibody (1∶500; catalog number: MAB360; Millipore), mouse monoclonal anti-Iba1 antibody (1∶100; catalog number: ab15690; Abcam) and mouse monoclonal anti-NeuN antibody (1∶500; catalog number: MAB377; Millipore). After being incubated with the primary antibodies at 4°C overnight, sections were washed in TBS with 0.025% triton-X 100. The donkey anti-mouse IgG (H+L) antibody conjugated with the fluorochrome NL493 (1∶200; catalog number: NL009; R&D Systems) and the donkey anti-rabbit IgG (H+L) antibody conjugated with the fluorochrome NL557 (1∶200; catalog number: NL004; R&D Systems) were applied for 1 h at room temperature. After washed in TBS, sections were mounted and coverslipped with the aid of Vectashield mounting medium (catalog number: H-1000; Vector labs, Burlingame, CA). Images were acquired with a fluorescence microscope with a CCD camera. Fluorescent intensity of Iba1 staining in the dentate gyrus area was measured by Image J software. Density of 7–12 randomly selected fields in the dentate gyrus of each side of hippocampus was averaged to reflect the value of each rat. This quantification was performed by a person who was blind to the group assignment of animals.

### Statistical Analysis

Results are presented as means±S.D. (n≥4). The results from the training sessions of Barnes maze and fear conditioning test were analyzed by two-way (isoflurane vs. no-isoflurane exposure) repeated measures analysis of variance followed by the Student-Newman-Keuls test. Other results were tested by two-way analysis of variance, by one way analysis of variance followed by the Student-Newman-Keuls test after confirmation of normal distribution of the data, by Kruskal-Wallis analysis of variance on ranks followed by the Student-Newman-Keuls when the data are not normally distributed, or by Student’s t test as appropriate. A P≤0.05 was accepted as significant. All statistical analyses were performed with the SigmaStat (Systat Software, Inc., Point Richmond, CA, USA).
